# Clinical Outcomes of Transcatheter Aortic Valve Replacement in Nonagenarians: A Systematic Review and Meta-Analysis

**DOI:** 10.1155/2019/5819232

**Published:** 2019-02-24

**Authors:** Yan Liu, Yu Du, Mingjie Fu, Yue Ma, Deguang Wang, Jinglin Zhang, Wei Liu, Yingxin Zhao, Yujie Zhou

**Affiliations:** Department of Cardiology, 12th ward, Beijing Anzhen Hospital, Capital Medical University, Beijing Institute of Heart Lung and Blood Vessel Disease, Beijing Key Laboratory of Precision Medicine of Coronary Atherosclerotic Disease, Clinical Center for Coronary Heart Disease, Beijing 100029, China

## Abstract

**Objectives:**

To compare the incidence of mortality and complications between nonagenarians and younger patients undergoing transcatheter aortic valve replacement (TAVR).

**Background:**

TAVR has become an alternative treatment for nonagenarian patients with severe aortic stenosis. Previous studies have reported conflicting results regarding the clinical outcomes between nonagenarians and younger patients who underwent TAVR.

**Methods:**

We searched PubMed, EMBASE, and Cochrane Library databases with predefined criteria from the inception dates to July 8, 2018. The primary clinical endpoint was 30-day and 1-year all-cause mortalities. Secondary outcomes were considered the rates of stroke, myocardial infarction, any bleeding, any acute kidney injury, any vascular complications, new pacemaker implantation, and conversion to surgical aortic valve replacement.

**Results:**

A total of 5 eligible studies with 25,371 patients were included in this meta-analysis. Compared with younger patients who underwent TAVR, nonagenarians had a significantly higher mean Society of Thoracic Surgeons score (STS score) (MD, 2.80; 95%CI: 2.58, 3.30; P<0.00001) and logistic European System for Cardiac Operative Risk Evaluation (logistic EuroSCORE) (MD, 2.72; 95%CI: 1.01, 4.43; P=0.002). Nonagenarians were associated with significantly higher 30-day mortality (6.2% vs. 3.7%; OR, 1.73; 95%CI: 1.49, 2.00) and 1-year mortality (15.5% vs. 11.8%; OR, 1.39; 95%CI: 1.26, 1.53), without significant statistical heterogeneity. Nonagenarians were associated with significantly increased rates of major or life-threatening bleeding, vascular complications and stroke of 20%, 35%, and 32%, respectively. There were no significant differences in the rate of myocardial infarction, stage 2 or 3 acute kidney injury, new pacemaker implantation, or conversion to surgical aortic valve replacement.

**Conclusions:**

Nonagenarians showed worse clinical outcomes than younger patients after TAVR, while the incidence of mortality was acceptable. TAVR remains an option for nonagenarian patients with severe aortic stenosis and should be comprehensively evaluated by the heart valve team.

## 1. Introduction

As forecasted by Kontis et al., the life expectancy in industrialized countries would break the 90-year barrier by 2030 [1]. Since the prevalence of aortic stenosis is increasing with lifespan, [2, 3] it is urgent to address the management of aortic stenosis (AS) for nonagenarians.

Since 2002, when the first transcatheter aortic valve replacement (TAVR) was carried out by Cribier, [4] it has become an alternative treatment for intermediate- to high-risk patients with severe AS [5, 6]. Several studies have shown no significant differences in the short- or long-term survival of nonagenarians between surgical aortic valve replacement (SAVR) and TAVR [7–9]. In consideration of frailty and inoperability, TAVR is a preferable approach for elderly patients.

However, previous studies have reported conflicting results regarding the clinical outcomes between nonagenarians and younger patients who underwent TAVR [10–14]. To further confirm the feasibility and safety of TAVR in nonagenarians, we performed this systematic review and meta-analysis to explore the short- to mid-term clinical outcomes of TAVR.

## 2. Methods

This meta-analysis was performed based on the Cochrane Handbook for Systematic Reviews of Interventions [15] and is presented according to the MOOSE (Meta-analysis of Observational Studies in Epidemiology) guidelines [16].

### 2.1. Search Strategy

We searched PubMed, EMBASE, and Cochrane Library databases for the current literature. The following search terms were used: (((((“transcatheter aortic valve replacement"[mesh]) OR transcatheter aortic valve implantation) OR TAVR) OR TAVI)) AND (((“Aged, 80 and over"[mesh]) OR 90 years) OR Nonagenarian*∗*). No language, publication date, or publication status restrictions were imposed. The last search was performed on July 8, 2018. Two investigators (YL and YD) performed the initial search separately, deleted duplicate records, screened titles and abstracts for relevance, and identified relevant articles for further full-text assessment. Reference lists from these retrieved articles were manually screened to identify additional relevant studies.

### 2.2. Inclusion Criteria

Studies were selected based on the following inclusion criteria: (1) studies enrolling nonagenarian and younger patients undergoing TAVR in current clinical practices, (2) studies comparing clinical outcomes of nonagenarians to younger patients undergoing TAVR, and (3) studies in which the clinical endpoints and adverse events were diagnosed by the Valve Academic Research Consortium 2 definitions [17]. Conference abstracts, reviews, comments, and editorials were excluded.

### 2.3. Data Extraction and Quality Assessment

Two investigators (YL and YD) independently extracted data (first author, country of origin, publication year, number of enrolled patients, and baseline patient characteristics) using a standardized data abstraction form. When the same patients were reported in several publications, only the largest study was used for the meta-analysis to avoid data duplication.

Two investigators (YL and YD) independently assessed the quality of selected studies based on the 9-star Newcastle-Ottawa Scale [18]. This scale rates studies based on eight criteria in three sources of bias. Disagreement was resolved by discussions and by consulting a third investigator (YJZ).

### 2.4. Clinical Endpoints

The primary clinical endpoint of interest was 30-day and 1-year all-cause mortality, and secondary outcomes were considered as the rates of stroke, myocardial infarction, any bleeding, any acute kidney injury, any vascular complications, new pacemaker implantation, and conversion to surgical aortic valve replacement. All definitions of clinical endpoints were based on the Valve Academic Research Consortium 2 definitions.

### 2.5. Statistical Analysis

The odds ratios (ORs) with 95% confidence intervals (CIs) for the endpoints were calculated from each study. Trial-specific ORs were combined with the Mantel-Haenszel fixed-effects model or with random effects model if heterogeneity was statistically significant or I^2^ > 50%. If no events were reported for one group in a comparison, a value of 0.5 was added to both groups for each of these studies. Trials with no events in both groups were not included in the meta-analysis when the ORs were calculated.

The presence of heterogeneity among studies was evaluated with the Cochran Q chi-squared test, with P<0.10 considered to indicate statistical significance, and the I^2^ test was used to evaluate inconsistencies. The I^2^ statistic is derived from the Q statistic and describes the percentage of total variation across studies which is due to heterogeneity; values of 25%, 50%, and 75% correspond to low, moderate, and high heterogeneity, respectively. The funnel plot was not drawn for outcomes due to the small number of studies included in this analysis.

We did not contact the authors of the included studies to obtain raw data. All analyses were performed using Review Manager version 5.3 (Copenhagen, Denmark; Cochrane Collaboration). All tests were two tailed, and P< 0.05 was considered significant.

## 3. Results

### 3.1. Study Characteristics and Quality Assessment

**Figure 1:**
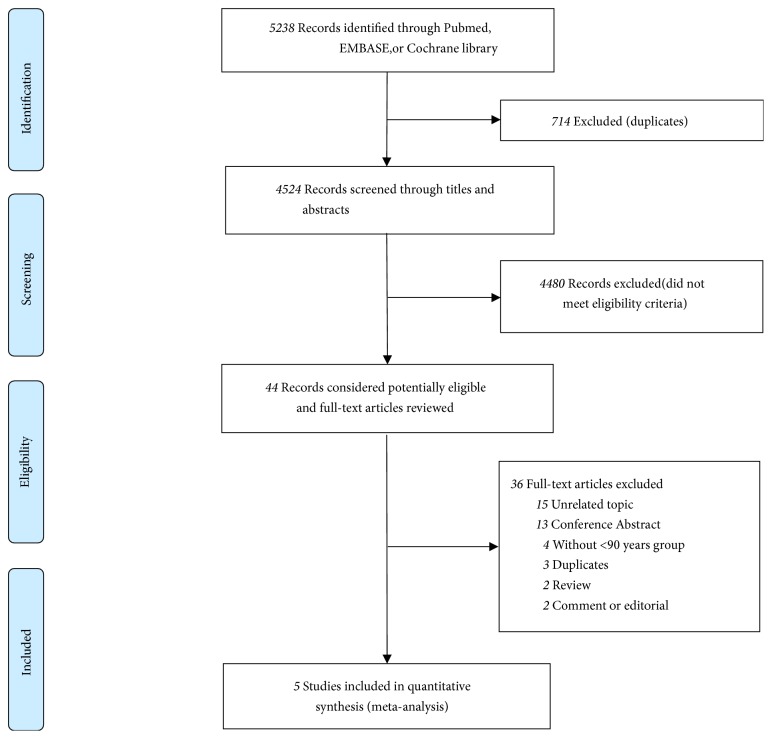
Flow diagram of study selection process.

From the searches for meta-analysis, 5,238 potentially eligible studies were identified. Titles and abstracts of these studies were screened for inclusion. Full-texts of 44 studies were read, and 5 studies met the inclusion criteria (Figure 1) [10, 19–22]. The main characteristics of the included studies are described in Table 1(a). The current meta-analysis included 25,371 patients (3,929 in the nonagenarian group and 21,442 in the younger group). The mean Society of Thoracic Surgeons score (STS score), logistic European System for Cardiac Operative Risk Evaluation (logistic EuroSCORE), left ventricular ejection fraction (LVEF), mean pressure gradient, and medical history of the different studies are summarized in Tables 1(b) and 1(c). All trials reported that the clinical outcomes of interest varied from a 30-day to a 3-year follow-up period.

The assessment of quality is presented in Table 2. The total score of the 5 observational studies was >5 according to the Newcastle-Ottawa Scale for risk of bias in observational studies, representing a low risk of bias.

### 3.2. Clinical Outcomes

**Figure 2:**
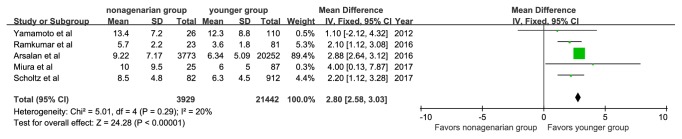
Forest plot of pooled analysis comparing Society of Thoracic Surgeons score of nonagenarians versus younger patients.

**Figure 3:**
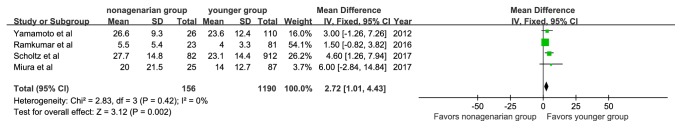
Forest plot of pooled analysis comparing logistic European System for Cardiac Operative Risk Evaluation of nonagenarians versus younger patients.

Nonagenarians had a significantly higher mean STS score than younger patients (MD, 2.80; 95%CI: 2.58, 3.30; P<0.00001), with low study heterogeneity (P=0.29; I^2^=20%, Figure 2). Four studies analyzed the logistic EuroSCORE of patients, which was also higher in the nonagenarian group (MD, 2.72; 95%CI: 1.01, 4.43; P=0.002). No significant statistical heterogeneity was found among the studies (Figure 3).

**Figure 4:**
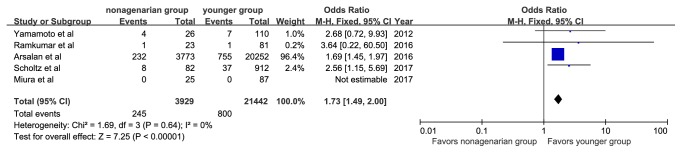
Forest plot of pooled analysis comparing 30-day mortality of nonagenarians versus younger patients.

**Figure 5:**
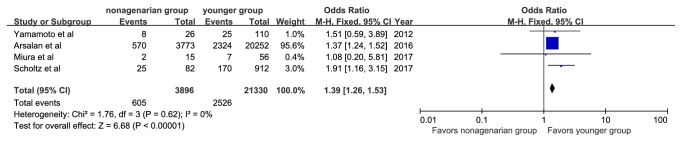
Forest plot of pooled analysis comparing 1-year mortality of nonagenarians versus younger patients.

There were 245 patients (6.2%) with 30-day mortality reported among the nonagenarian group and 800 patients (3.7%) among the younger group. The 30-day mortality rates were significantly higher among nonagenarians (OR, 1.73; 95%CI: 1.49, 2.00; I^2^=0%, Figure 4). Four studies reported 1-year all-cause mortalities. The pooled average 1-year mortality was 12.4% and was 15.5% in the nonagenarian group and 11.8% in the younger group. Nonagenarians were associated with a significantly higher 1-year mortality (OR, 1.39; 95%CI: 1.26, 1.53; I^2^=0%, Figure 5).

**Figure 6:**
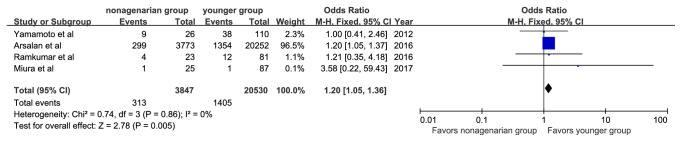
Forest plot of pooled analysis comparing the rates of major or life-threatening bleeding in nonagenarians versus younger patients.

**Figure 7:**
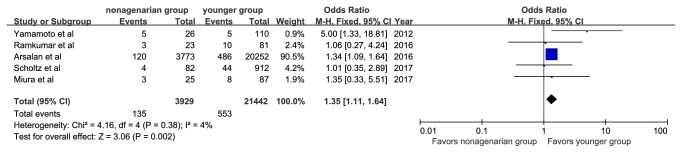
Forest plot of pooled analysis comparing the rates of vascular complication in nonagenarians versus younger patients.

**Figure 8:**
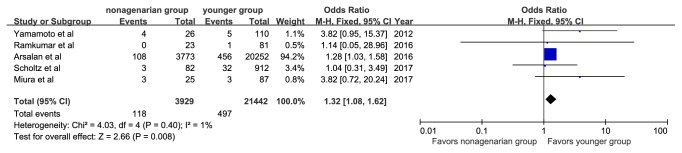
Forest plot of pooled analysis comparing the rates of stroke in nonagenarians versus younger patients.

The specific definitions of any bleeding, any acute kidney injury, and any vascular complications are shown in Table 3. Major or life-threatening bleeding was reported in 313 patients (8.1%) in the nonagenarian group and in 1,405 patients (6.8%) in the younger group. Vascular complications were reported in 135 patients (3.43%) in the nonagenarian group and 553 (2.6%) in the younger group. Nonagenarians were associated with a significantly higher rate of major or life-threatening bleeding (OR, 1.20; 95%CI: 1.05, 1.36; I^2^=0%, Figure 6) and vascular complications (OR, 1.35; 95%CI: 1.11, 1.64; I^2^=4%, Figure 7). In addition, we observed that nonagenarians had a higher risk of stroke than younger patients, with evidence of low heterogeneity (OR, 1.32; 95%CI: 1.08, 1.62; I^2^=1%, Figure 8).

**Figure 9:**
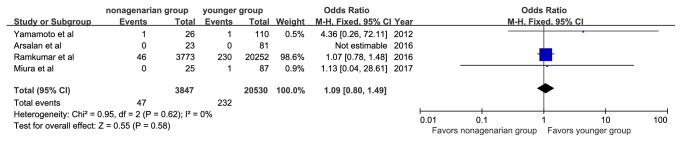
Forest plot of pooled analysis comparing the rates of myocardial infarction in nonagenarians versus younger patients.

**Figure 10:**
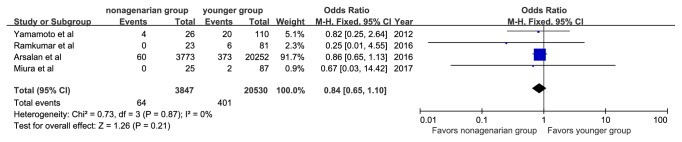
Forest plot of pooled analysis comparing the rates of stage 2 or 3 acute kidney injury in nonagenarians versus younger patients.

**Figure 11:**
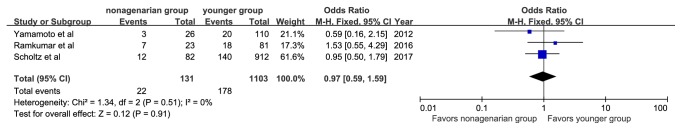
Forest plot of pooled analysis comparing the rates of new pacemaker implantation in nonagenarians versus younger patients.

**Figure 12:**
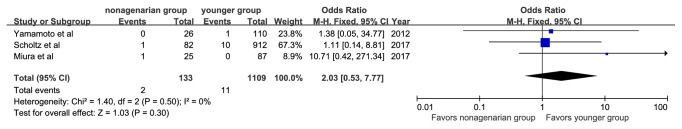
Forest plot of pooled analysis comparing the rates of conversion to aortic valve replacement in nonagenarians versus younger patients.

There were no significant differences in the rate of myocardial infarction (OR, 1.09; 95%CI: 0.80, 1.49; I^2^=0%), stage 2 or 3 acute kidney injury (OR, 0.84; 95%CI: 0.65, 1.10; I^2^=0%), new pacemaker implantation (OR, 0.97; 95%CI: 0.59, 1.59; I^2^=0%), or conversion to surgical aortic valve replacement (OR, 2.03; 95%CI: 0.53, 7.77; I^2^=0%) (Figures 9–12).

## 4. Discussion

To the best of our knowledge, this is the first comprehensive review of the current literature comparing the clinical outcomes of TAVR between nonagenarian and younger patients in a meta-analytic approach. In the results reported here, nonagenarians, with a higher mean STS score and logistic EuroSCORE, had an increased 30-day and 1-year postoperative all-cause mortalities compared with the younger group. In addition, the rates of major or life-threatening bleeding, vascular complications and stroke were also higher in nonagenarians. Furthermore, no significant differences were observed in the rates of myocardial infarction, stage 2 or 3 acute kidney injury, new pacemaker implantation, and conversion to surgical aortic valve replacement between the two groups.

Although the mortality rate for nonagenarians in our study remained higher than for younger patients, considering life expectations, comparison of medical treatment, and quality of life, the mortality rate may be acceptable. It was reported in nonagenarians that age alone accounted for a predicted logistic EuroSCORE mortality risk of 6.55% for male patients, and for female patients, this risk rises to 8.89% without any other preoperative risk factors [23]. Similarly, our results showed that nonagenarians had a significantly higher logistic EuroSCORE than younger patients (MD, 2.72; 95%CI: 1.01, 4.43). Bernal et al. indicated that approximately one-third of nonagenarians with severe aortic stenosis have few comorbidities [24]. Therefore, in a particular population, patients at a very advanced age could be the primary factor making nonagenarians have a high risk for surgery. Furthermore, Bernal et al. observed that nonagenarians who underwent conservative management tended to have a higher 1-year mortality than those who underwent TAVR (58% vs. 40.7%, P=0.097) [24]. In our meta-analyses, the pooled postoperative 30-day and 1-year all-cause mortalities of nonagenarians was 6.2% and 15.5%, respectively. Despite the higher mortality than younger patients, TAVR could be a better option for severe AS nonagenarians.

Manolis et al. demonstrated that, in nonagenarians who underwent TAVR, bleeding and vascular complications ranged from 9% to 34% (average 16%), and stroke risk ranged from 2% to 18% (average 3–4%) [25]. Similarly, in this meta-analysis, we indicated that nonagenarians were associated with significantly increased rates of major or life-threatening bleeding, vascular complications and stroke of 20%, 35%, and 32%, respectively. The higher incidence of vascular complications may be attributed to the higher rates of transfemoral access. Another possible reason could be the higher rates of vascular calcification [26] in the “oldest old” population. The higher incidence of stroke and major or life-threatening bleeding may be attributed to the higher prevalence of atrial fibrillation [11] and antiplatelet therapy after TAVR [27], respectively.

In the general population, life expectancy in nonagenarians is 2.5 and 3.5 years for men and women, respectively [23]. The rate of 1-year mortality in nonagenarians was 19.3% [28]. Accordingly, we should focus on the improvement of quality of life and try to add life to their years instead of years to their life [29]. Stanska et al. showed that the quality of life in elderly patients was significantly improved at the one-month follow-up after TAVR [30]. Mack et al. indicated that nonagenarians undergoing TAVR have an improvement in quality of life (47.2 to 74.0, P=0.051), as measured by the Kansas City Cardiomyopathy Questionnaire (KCCQ) [7]. Arsalan et al. reported that nonagenarians have similar KCCQ scores with younger patients 1 year after TAVR [10].

Nonagenarians referred for TAVR should be evaluated carefully by the heart valve team composited of cardiovascular surgeons, interventional cardiologists, imaging specialists, cardiovascular anesthesiologists, and cardiovascular nursing professionals [31]. Generally, we use the STS score and logistic EuroSCORE to assess the risk of TAVR, and neither of these scores includes the specific geriatric conditions, which tend to generate a considerable impact on prognosis in elderly patients. Okoh et al. indicated that frailty status is independently associated with increased mortality after TAVR (hazard ratio: 1.84; 95%CI: 1.06–3.17; P=0.028) [32]. Multidimensional Geriatric Assessment (MGA) is a diagnostic process that determines the medical and functional resources and problems of elderly patients. According to Stortecky et al., risk prediction of TAVR can be improved by adding MGA-based information to global risk scores [33]. Furthermore, in nonagenarians, transapical TAVR was associated with a significantly higher risk of early mortality compared with transfemoral TAVR [34–37]. The procedure should be comprehensively evaluated by the heart valve team.

This study has several limitations. First, all of the eligible studies were observational studies, and the results may have been affected by unmeasured confounding variables. Second, due to the limited number of studies included in this analysis, we did not conduct sensitivity and meta-regression analysis for outcomes. Third, our analysis did not include individual patient data. Finally, these data are mostly from highly experienced TAVR centers and may not be generalizable to other hospitals with less experience.

In conclusion, the results reported here suggest that nonagenarians showed higher short- to mid-term mortalities and higher rates of major or life-threatening bleeding, vascular complications and stroke compared to younger patients. However, the rate of mortality in nonagenarians is potentially acceptable. TAVR remains an optional therapy for nonagenarian patients, which should be comprehensively evaluated by the heart valve team.

## Figures and Tables

**Table:** (a) Baseline characteristics of the included studies

First Author	Country	Year	Multicenter	Number of Patient		Mean Age (years)		Male (%)	
				nonagenarian group	control group	nonagenarian group	control group	nonagenarian group	control group
Yamamoto et al.	France	2012	No	26	110	91.6±1.9	82.3±7.0	19	50
Arsalan et al.	United States	2016	Yes	3773	20252	92.0 (90.0-93.0)	82.0 (76.0-86.0)	48.24	50.74
Ramkumar et al.	Australia	2016	No	23	81	90.6±2.6	81.1±4.6	52	44
Miura et al.	Japan	2017	No	25	87	91.6±1.7	82.5±6.0	20	37.9
Scholtz et al.	Germany	2017	No	82	912	91.8±1.4	84.8±2.6	19.5	58.8

**Table:** (b) Baseline characteristics of patients in the included studies

First Author	Mean STS (%)		Logistic EuroSCORE		Left Ventricle EF (%)		Mean Gradient (mm Hg)	
	nonagenarian group	control group	nonagenarian group	control group	nonagenarian group	control group	nonagenarian group	control group
Yamamoto et al.	13.4±7.2	12.3±8.8	26.6±9.3	23.6±12.4	51.3±12.3	48.6±14.2	56.3±23.4	45.5±15.4
Arsalan et al.	9.22 (6.73-13.25)	6.34 (4.20-9.77)	NR	NR	58(48-65)	56(45-63)	NR	NR
Ramkumar et al.	5.7±2.2	3.6±1.8	5.5±5.4	4.0±3.3	53.7±17	59.1±11	44.7±14	49.6±16
Miura et al.	10.0(7.5-12.0)	6.0(3.0-7.0)	20.0(16.0-25.5)	14.0(11.0-22.0)	63.2±6.9	61.8±9.0	59.6±19.0	54.2±19.9
Scholtz et al.	8.5±4.8	6.3±4.5	27.7±14.8	23.1±14.4	54.6±9.5	52.5±11.4	47.1±17.3	45.1±16.8

**Table:** (c) Medical history of patients in the included studies

First Author	Hypertension (%)		Diabetes Mellitus (%)		CAD (%)		Stroke (%)		NYHA III-IV (%)		COPD (%)	
	nonagenarian group	control group	nonagenarian group	control group	nonagenarian group	control group	nonagenarian group	control group	nonagenarian group	control group	nonagenarian group	control group
Yamamoto et al.	73	76	23	25	NR	NR	NR	NR	65	64	19	30
Arsalan et al.	86.56	89.26	19.35	40.39	NR	NR	9.33	12.77	80.97	81.37	15.4	30.1
Ramkumar et al.	57	81	14	26	22	20	9	17	74	69	27	23
Miura et al.	72	71.3	4	26.4	20	44.8	4	15.1	72	63.2	12	17.2
Scholtz et al.	82.9	79.6	11	22.1	50.1	57	NR	NR	NR	NR	15.9	14.5

CAD: coronary artery disease; COPD: chronic obstructive pulmonary disease.

**Table 2:** Newcastle-Ottawa Scale of the included studies.

Study	Selection				Comparability	Outcome			Total Stars
	Exposed Cohort	Non-Exposed Cohort	Ascertainment of Exposure	Outcome of Interest		Assessment of Outcome	Length of Follow-Up	Adequacy of Follow-Up	
Yamamoto, 2012	*∗*	*∗*	*∗*	*∗*	*∗*	*∗*	*∗*	*∗*	8
Arsalan, 2016	*∗*	*∗*	*∗*	*∗*	*∗*	/	*∗*	*∗*	7
Ramkumar, 2016	*∗*	*∗*	*∗*	*∗*	*∗*	/	*∗*	*∗*	7
Miura, 2017	*∗*	*∗*	*∗*	*∗*	*∗*	/	*∗*	*∗*	7
Scholtz, 2017	*∗*	*∗*	*∗*	*∗*	*∗*	/	*∗*	*∗*	7

**Table 3:** The specific definitions of bleeding, acute kidney injury, and vascular complications.

First Author	Bleeding	Acute kidney injury	vascular complications
Yamamoto et al.	Major, life-threatening	Stage 2 or 3	Major
Arsalan et al.	Major	New requirement for dialysis	Major, minor
Ramkumar et al.	Major	Stage 2 or 3	Major
Miura et al.	Life-threatening	Stage 2 or 3	Major, minor
Scholtz et al.	NR	NR	No specified

## Data Availability

The data supporting this article are from previously reported studies and datasets, which have been cited. All relevant data are within the article.

## Abbreviations

TAVR: Transcatheter aortic valve replacement
STS score: Thoracic Surgeons score
logistic EuroSCORE: Logistic European System for Cardiac Operative Risk Evaluation
AS: Aortic stenosis
SAVR: Surgical aortic valve replacement
MOOSE: Meta-analysis of Observational Studies in Epidemiology
OR: Odds ratio
CI: Confidence interval
LVEF: Left ventricular ejection fraction
KCCQ: Kansas City Cardiomyopathy Questionnaire
MGA: Multidimensional Geriatric Assessment
CAD: Coronary artery disease
COPD: Chronic obstructive pulmonary disease.
